# Gratitude and Adaptive Coping Among Chinese Singaporeans During the Beginning of the COVID-19 Pandemic

**DOI:** 10.3389/fpsyt.2020.628937

**Published:** 2021-01-26

**Authors:** Eddie M. W. Tong, Vincent Y. S. Oh

**Affiliations:** Department of Psychology, National University of Singapore, Singapore, Singapore

**Keywords:** gratitude, COVID-19, coping, health behavioral intention, Chinese

## Abstract

We report results of a cross-sectional survey conducted during March–April 2020 which marked the start and escalation of the COVID-19 crisis in Singapore. Our purpose was to examine whether reported feelings of gratitude among Chinese Singaporeans (*N* = 371; 124 males, 247 females; *M*_*age*_ = 22.54, *SD*_age_ = 3.63, age range: 18–53 years) could be linked to adaptive responses to the pandemic. The results revealed that gratitude was associated with stronger endorsement of virus-prevention measures (β = 0.25, *p* = 0.001) that are necessary for protecting the physical health of oneself and others but disruptive to daily lives. Gratitude was also positively related to the tendency to perceive meaningful benefits in the crisis (β = 0.25, *p* = 0.002). Importantly, demonstrating the uniqueness and robustness of gratitude as a predictor of positive coping in response to the pandemic, these relationships remained significant when controlling for other protective psychological factors (resilience and optimism), emotions, and key demographic variables. Among the emotions measured, gratitude was also reported the most strongly. The findings support theoretical models that gratitude facilitates prosocial inclinations and openness to different ways to support the well-being of others and suggest that in a collectivistic culture, gratitude could be a key resource enabling adaptation to a crisis.

## Introduction

Gratitude is a positive emotional response to receiving a positive outcome from another person. It inspires the recipient to be prosocial (1, 2) and brings about positive outcomes such as lower maladjustment and higher well-being (3). However, much less is known about the roles that gratitude might play in a major crisis such as the current COVID-19 pandemic, which as of this writing has infected over 44 million people worldwide and taken the lives of over a million victims (4). A question in emotion research is whether positive emotions, and gratitude in particular, could continue to function as a protective factor to support adjustment and maintain well-being in a calamity of this severity. In this paper, we report the first study that examined the relationships between gratitude and endorsement of virus-prevention measures and benefit-finding in the early stages of the COVID-19 pandemic among a sample of Chinese Singaporeans.

Why might investigating the protective function of gratitude specifically (1) during early stages of the pandemic and (2) among the Chinese be important? Gratitude is known to predict better coping and adjustment (5, 6). For instance, it predicted adjustment among Vietnam War veterans with post-traumatic stress disorder (PTSD) symptoms (7). Among Israeli survivors of missile attacks, gratitude was negatively associated with PTSD symptoms 2.5 months after the attacks (8). Studies have also shown that gratitude is associated with lower burnout (9), suicide risk (10), and depression (6) in non-crisis contexts. However, none of these studies have examined an international calamity like the COVID-19 pandemic. When it emerged in the first half of 2020, scientists and laypersons did not fully comprehend the virus other than that it appeared highly infectious and more fatal than the common flu. There was the ominous foreboding that the virus would put not only the lives of millions worldwide at dire risk but also their livelihood at jeopardy, with no end in the form of a vaccine in sight. Exacerbating the uncertainty is misinformation concerning the virus and alternative practices (11). It is pertinent to ask whether or not the usual protective factors (including gratitude) known to enable adjustment under normal contexts would function just as effectively in a poorly understood crisis that COVID-19 still is.

Chinese people refer to those associated with China based on ancestry, ethnicity, or nationality. Chinese nationals and ethnic Chinese born outside of China comprise about 18% of the world population (12–14). Yet, few studies have been done on how the Chinese—the largest ethnic group in the world—respond to the pandemic. Furthermore, controlling the pandemic would minimally require people to behave responsibly by practicing safe health behaviors to reduce spreading of the virus. If gratitude—a positive socially oriented emotion—has any effect in promoting these other-focused behaviors, it should be found among the Chinese who tend to endorse collectivistic values. Focusing on Chinese samples is thus a major first step to test the protective function of gratitude in response to an impending crisis.

Theoretical grounds for understanding the protective functions of gratitude can be based on Fredrickson's (15) broaden-and-build theory, which states that positive emotions broaden cognitive and behavioral abilities in the short-run and build them into stable tendencies in the long-run. Her model posits a similar process for gratitude (16). Gratitude could have two short-term effects. It may inspire prosocial responses on a daily basis, nudging one to be sensitive to others and motivating helpful behavior. There is robust evidence that gratitude facilitates prosociality (17). In addition, gratitude may regularly enhance the ability to make mental shifts. Researchers have theorized that grateful people could be driven by their prosocial desires toward thinking of different ways to help people (16, 18), thus promoting an agile mindset that is receptive to diverse ideas. Temporal accumulation of these momentary broadening of prosocial motives and mental shifting can build over time to create stable prosocial tendencies and cognitive openness. That is, individuals who experience gratitude on a regular basis may become socially conscious individuals with flexible processing capacities that are open to new ways of helping others and supporting the community (16).

We posit that gratitude plays a role in enabling adaptive responses during early stages of the COVID crisis because managing the pandemic then demanded virus-prevention measures that require prosocial proclivity and cognitive openness (16, 18). These measures included regular hand washing, mask wearing, disinfecting belongings, avoiding hand-face contact, and social distancing. They are meant not just to protect oneself from the virus, but also to prevent an infected person from spreading the virus to others. They are not unusual practices—we observe them when having the common cold. However, as the crisis unfolded, it became increasingly clearer that the measures would have to be engaged habitually for a protracted period. This would mean upending daily routine, curtailing social activities, and even compromising businesses and careers because of social distancing. Hence, stopping the virus requires each person to behave responsibly to keep everyone else safe when doing so brings personal costs. Accordingly, those with greater prosocial intention should be more willing to adhere to the measures (1). At the same time, a good degree of openness and flexibility is needed. Some people resisted these measures given the major disruptions of lifestyle and livelihood they could bring. Demonstrations happened in some nations after their government mandated some of these measures. In addition, in the initial phase of the pandemic, it was not clear to some people whether some of these measures are effective and necessary. For instance, WHO encouraged mask-wearing for the general public only in mid-2020 because it was only then that scientific evidence for it became clear (19). Hence, individuals who are more open should be more willing to endorse the health-protecting but difficult measures. Given that gratitude facilitates prosocial motives and cognitive openness, we hypothesized gratitude to be positively related to the willingness to endorse these virus-prevention measures in early stages of the pandemic.

In addition, research has shown that gratitude is associated with an enhanced ability to find meaning and purposes even in abject situations. This relationship could be due to the greater cognitive flexibility posited of gratitude. For instance, gratitude prospectively predicted greater sense of coherence, mediated by positive reappraisal (20). Positively appraising events explained the negative association between gratitude and depression (21). Gratitude interventions have also been found to enable disengagement from negative cognitions (22). However, again, many studies were conducted in fairly normal circumstances, and whether gratitude will have similar effects in a pandemic is unknown. We hypothesized that gratitude should be positively associated with benefit-finding.

In sum, we predicted that gratitude should be associated with greater willingness to endorse socially responsible virus-prevention measures and benefit-finding during the early stages of COVID-19 pandemic. We report a study to test these hypotheses that was conducted in Singapore among ethnic Chinese Singaporeans during March–April 2020 when the pandemic began to escalate. Importantly, we also tested whether gratitude would robustly predict these outcomes over and above other potential predictors. Resilience and optimism have been found to predict mental health and the use of health-protective behavior (23–25). Hence, it is critical to examine whether gratitude would remain independently predictive of the outcomes controlling for them. They also included other emotions (specifically anger, sadness, anxiety, joy, pride, and care) which served the overall purpose of testing the uniqueness of gratitude. There is no direct relationship between negative emotions and health outcomes as much depends on how the negative feelings are regulated (26). Hence, we made no prediction on whether anger, sadness, anxiety would predict endorsement of virus-prevention measures and finding benefits. Controlling for joy would rule out the possibility that any protective function of gratitude is due only to its positive valence. Pride can elicit self-determined responses such as persistence (27) and hence might enable better coping. An ensuing question was whether gratitude might predict the outcomes independently of pride. Finally, care refers to general positive feelings of concern. Like gratitude, it is a positive emotion that is socially focused. However, it is unclear whether care also engenders the cognitive openness as gratitude does that encourages the use of new behavioral responses. If gratitude predicts endorsement of virus-prevention measures and benefit-finding independently of care, it would suggest that gratitude is unique among positive social emotions as a protective resource in handling the crisis. Finally, we included several demographic variables available in our dataset (namely, age, gender, education, household income, and household size) as predictors. We also coded the number of cumulative infections on the day of participation to account for whether the severity of the pandemic would affect how people respond. Whether or not these variables would predict endorsement of virus-prevention measures and benefit-finding is interesting in itself—to which we make no prediction of—but the pertinent issue is whether gratitude would remain predictive of the outcomes independently of these variables.

## Materials and Methods

### Participants

Four hundred and seventeen Chinese participants from Singapore were examined in this study. The survey was open to any Singaporean citizens above 18 years of age. Online advertisements were used to recruit participants, who were told that the study was interested in examining how they were managing the pandemic and which advertised a lucky draw of two $100 Singapore Dollar (SGD) prizes. The study consists of a cross-sectional survey which was conducted in Singapore between 26th March (683 cumulative infections) and 20th April (8,014 cumulative infections), during a time when the pandemic was increasingly escalating. Participants provided informed consent and were assured that their responses would be confidential and anonymized—identifying information (names and email addresses) was collected only on a separate survey for administering the lucky draw and was delinked from the main survey. Forty-six participants were excluded for failing attention checks, giving a final sample of 371 participants (124 males, 247 females; *M*_*age*_ = 22.54, *SD*_age_ = 3.63, age range: 18–53 years). Excluded participants generally did not differ from included ones in age, income, and education level (*p*s > 0.30) but were more likely to be male (*r* = 0.15, *p* = 0.002). Exclusion was also uncorrelated with any of the key predictor or outcome variables (*p*s > 0.05) except anger (*r* = 0.13, *p* = 0.006). Overall, included and excluded participants generally did not differ substantially, and any differences that did occur are relatively small and unlikely to affect the analyses. This study is approved by the National University of Singapore Institutional Review Board.

### Measures

#### Emotions

Participants were asked to refer to the ongoing COVID-19 virus outbreak and were given the following prompt: “Over the past two weeks, to what extent have you felt the following emotions as a result of this outbreak?” They rated several emotion items presented in randomized order on a seven-point scale, with the following anchors: 1 (“Did not feel the emotion at all”), 4 (“Felt the emotion moderately”), and 7 (“Felt the emotion very much”). Four positive emotions and three negative emotions were assessed. *Gratitude* was measured by two items (“Grateful,” “Thankful”; α = 0.86); pride was measured by two items (“Proud,” “Confident”; α = 0.59); care was measured by two items (“Love,” “Compassion”; α = 0.64); and joy was measured by two items (“Joyful,” “Happy”; α = 0.82). Sadness was measured by four items (“Sad,” “Lonely,” “Helpless,” “Hopeless”; α = 0.75); anger was measured by four items (“Angry,” “Hostile,” “Irritated,” “Contempt”; α = 0.73), and anxiety was measured by two items (“Fearful,” “Anxious”; α = 0.78). The internal consistency of several subscales were only moderate due to the small number of items, for which Cronbach's alpha often underestimates reliability (28, 29), and factor analytic evidence is recommended to provide stronger evidence of scale reliability (30). Confirmatory factor analyses supported the above emotion classifications; model fit was strong, χ^2^ (114) = 303.54, *p* < 0.001, CFI = 0.93, RMSEA = 0.067, SRMR = 0.054, and all items loaded into their respective factors strongly (standardized λs > 0.40)^1^.

#### Resilience

Six items adapted based on the Brief Resilience Scale [BRS (31)] assessed participants' resilience with regard to the COVID-19 outbreak (“I believe that I will bounce back quickly from the current crisis,” “I will have a hard time making it through the current crisis,” “It will not take me long to recover from the current crisis,” “It will be hard for me to snap back from the effects of the current crisis,” “I will come through this difficult crisis with little trouble,” “I will take a long time to get over the set-back caused by the current crisis.”) on a seven-point scale from 1 (“Strongly Disagree”) to 7 (“Strongly Agree”). Three items were reverse-coded, and the six items were then averaged (α = 0.86).

#### Optimism

Four items measured the extent to which participants were optimistic about the COVID-19 outbreak (“I believe the COVID-19 outbreak will be resolved successfully,” “I am confident that life will go back to normal soon,” “I am certain that the COVID-19 outbreak is manageable,” “I trust that we will be able to overcome the COVID-19 outbreak.”) on a seven-point scale from 1 (“Strongly Disagree”) to 7 (“Strongly Agree”). The four items were averaged (α = 0.85).

#### Virus-Prevention Measures

Participants were asked to rate how much they intend to follow seven virus-prevention measures involving social distancing and maintaining personal hygiene (e.g., “Wash your hands with soap or hand sanitizer frequently,” “Avoid touching your face,” “Avoid leaving your house except when necessary (e.g., when groceries run out),” “Regularly disinfect your belongings,” “Shower upon arriving home from outside,” “Minimize unnecessary social contact, such as social gatherings or sharing food with others,” “Wear a surgical mask if going out.”) which help to minimize the risk of being infected. The items were rated on a seven-point scale, with the following anchors: 1 (“Not at all likely to do this”), 4 (“Somewhat likely to do this”), and 7 (“Very likely to do this”). The seven items were averaged (α = 0.77).

#### Benefit-Finding

Five items adapted from Fredrickson et al. (32) measured benefit-finding from a crisis (“Do you feel that anything good would come out of dealing with the crisis?” “Do you feel that you might find benefit from this crisis in the long-term?” “Do you think it is likely that there is something to learn from this crisis?” “Do you think you would try to see the good side of the crisis?” “Do you think the crisis could change your life in a positive way?”) on a seven-point scale from 1 (“Not at all”) to 7 (“Very Much”). The five items were averaged (α = 0.82).

#### Covariates

We controlled for demographical variables, including age, gender (1 = “male,” 0 = “female”), education level (1 = “No school or some grade/primary school” to 11 = “Advanced degree beyond a Master's Degree”), annual household income (1 = “ < $10,000” to 8 = “$150,000 or more”), and household size. We also coded the cumulative number of infections on each participant's day of participation to control for the increasing severity of the crisis over time—due to the large numerical value of this variable, we further divided it by 100 to improve the interpretability of all regression coefficients.

#### Social Desirability

To control for the possibility that responses to some of the measures could be influenced by presentational concerns, we measured socially desirable tendencies using eight items from the Balanced Inventory of Desirable Responding [BIDR-16 (33)], of which four were reverse-coded. The items were rated on a seven-point scale from 1 (“Strongly Disagree”) to 7 (“Strongly Agree”). Following Hart et al. (33), each item was scored such that “6” or “7” were scored “1” while ratings below “6” were scored “0.” The eight scores were summed.

#### Checks

Two attention checks were administered to detect inattentive responses (e.g., “Maintaining good hygiene, but for this question select the option “2” to show that you are paying attention”).

## Results

These descriptive statistics are summarized in Table 1. Reported resilience (*M* = 4.96) and optimism (*M* = 5.21) were generally high, indicating that the sample on average was coping adaptively at the time of the study. Of note as well, specific emotions appeared to be more strongly activated. Unsurprisingly given the uncertainties of the crisis, anxiety (*M* = 3.95) is the most prevalent negative emotion reported. Interestingly, among the positive emotions, gratitude was the most strongly reported (*M* = 4.31), at above the midpoint of the scale (4 = “Felt the emotion moderately”). Joy (*M* = 2.49), pride (*M* = 3.01), and caring (*M* = 3.53) were not reported strongly.

**Table 1:** Descriptive statistics for all key variables.

	***M***	***SD***	**Range**
Number of cases during participation	1,436.00	1,292.34	683–8,014
Age	22.54	3.63	18–53
Gender	–	–	247 females (66.58%), 124 males (33.42%)
Education level	5.09	1.12	3–10
Household income	3.56	2.23	1–8
Household size	4.28	1.18	1–9
Social desirability	1.55	1.69	0–8
Resilience	4.96	1.08	1.67–7
Optimism	5.21	1.20	1.50–7
Gratitude	4.31	1.60	1–7
Joy	2.49	1.25	1–6.5
Pride	3.01	1.38	1–7
Caring	3.53	1.39	1–7
Sadness	3.19	1.26	1–7
Anxiety	3.95	1.43	1–7
Anger	2.96	1.19	1–7
Virus-prevention measures	4.86	1.21	1.29–7
Benefit-finding	4.85	1.16	1–7

*Education level was measured in continuous increasing order, with 1 representing “No school/some primary school” and 11 representing “Advanced degree beyond a Master's degree”. The mean of 5.09 approximates “Some undergraduate education, no degree (college or university).” Household income was measured in continuous increasing order with 1 representing “ < SGD$10,000” and 8 representing “SGD$150,000 or more.” The mean score of 3.56 approximates the range between “SGD$25,000–SGD$34,999” and “SGD$35,000–SGD$49,999”*.

The correlation matrix is provided in Table 2. As shown in Table 2, gratitude correlated positively with both endorsement of virus-prevention measures and benefit-finding and the effect sizes were medium. Next, to test whether gratitude predicted the outcome variables independently, we performed two hierarchical linear regressions predicting endorsement of virus-prevention measures and benefit-finding, with gratitude as the focal predictor which was entered at the second step. Resilience, optimism, anger, anxiety, sadness, joy, pride, and caring were included as comparisons to gratitude at the first step. Number of cases, age, gender, education level, household income, household size, and social desirability were controlled for in the first step as well. No evidence of multicollinearity emerged in any analyses (VIFs < 2.5), and *post-hoc* power analyses indicated very strong power of 0.90 for detecting small-to-medium effect sizes.

**Table 2:** Correlation matrix for all key variables.

	**1**	**2**	**3**	**4**	**5**	**6**	**7**	**8**	**9**	**10**	**11**	**12**	**13**	**14**	**15**	**16**	**17**
1. Cases	–																
2. Age	0.06	–															
3. Gender	−0.03	0.21^**^	–														
4. Education	0.08	0.32^***^	0.01	–													
5. HH income	−0.02	0.13^*^	−0.07	0.04	–												
6. hh size	−0.05	−0.04	−0.06	−0.12^*^	0.08	–											
7. SDS	0.05	0.06	0.04	0.08	−0.05	−0.04	–										
8. Resilience	−0.08	0.11^*^	0.07	0.07	0.11	−0.05	0.23^***^	–									
9. Optimism	−0.03	0.07	0.08	0.08	0.09	0.05	0.03	0.38^***^	–								
10. Gratitude	0.09	0.02	−0.01	0.02	0.07	0.06	−0.03	0.15^**^	0.20^***^	–							
11. Joy	0.11^*^	0.03	0.05	0.12^*^	0.04	−0.04	−0.02	0.03	0.15^**^	0.40^***^	–						
12. Pride	−0.01	0.03	0.17^**^	0.06	0.04	0.03	−0.08	0.12^*^	0.18^***^	0.58^***^	0.44^***^	–					
13. Caring	0.08	0.05	−0.01	0.09	0.17^**^	0.03	−0.02	0.05	0.10	0.61^***^	0.45^***^	0.52^***^	–				
14. Sadness	0.07	0.04	−0.11^*^	0.12^*^	0.05	0.06	−0.13^*^	−0.32^***^	−0.08	0.16^**^	0.15^**^	0.05	0.27^***^	–			
15. Anxiety	0.10	−0.01	−0.13^*^	0.08	0.04	0.08	−0.14^**^	−0.33^***^	−0.10	0.25^***^	0.08	0.08	0.27^***^	0.62^***^	–		
16. Anger	−0.01	−0.05	−0.04	−0.04	0.08	0.01	−0.11^*^	−0.27^***^	−0.04	0.18^***^	0.16^***^	0.17^**^	0.27^***^	0.53^***^	0.50^***^	–	
17. VPM	0.22^***^	0.02	−0.08	0.04	0.01	−0.02	0.06	−0.11	−0.01	0.23^***^	0.01	0.04	0.19^***^	0.21^***^	0.36^***^	0.15^**^	–
18. BF	0.01	0.02	−0.02	−0.01	0.10	−0.04	0.01	0.22^***^	0.28^***^	0.39^***^	0.20^***^	0.25^***^	0.30^***^	0.07	0.12^*^	0.03	0.18^**^

* *p < 0.05*,

** *p < 0.01*,

*** *p < 0.001. Gender was coded with “1” representing males and “0” representing females. HH, household; SDS, social desirability; VPM, virus-prevention measures; BF, benefit-finding*.

At the first step, the control variables explained significant variance in virus-prevention measures (*R*^2^ = 0.21, *p* < 0.001), but gratitude nevertheless explained additional variance when entered in the second step (Δ*R*^2^ = 0.03, *p* = 0.001). The full model significantly explained variance in virus-prevention measures, *F*(16, 264) = 5.23, *p* < 0.001, Adjusted *R*^2^ = 0.20. As shown in Table 3, gratitude remained predictive of greater endorsement of virus-prevention measures controlling for other predictors and the demographic variables. Optimism and resilience did not significantly predict the endorsement of these behaviors. Among the other emotions, only anxiety independently and positively predicted higher endorsement, while joy predicted lower endorsement. Anger, sadness, pride, and caring were not significant predictors. As shown in Table 2, there were significant positive relationships between anger, sadness, and care and endorsement but these relationships were not robust when controlling for gratitude and other predictors. None of the demographic predictors was associated with endorsement of the measures, but as the number of cases increased, participants were more likely to endorse the measures.

**Table 3:** Regression coefficients predicting virus-prevention measures and benefit-finding.

	**Virus-prevention measures**					**Benefit-finding**				
	***b***	***SE***	***p***	**β**	**95% CI**	***b***	***SE***	***p***	**β**	**95% CI**
Cases	0.02^**^	0.01	0.001	0.18	[0.01, 0.03]	−0.002	0.01	0.59	−0.03	[−0.01, 0.01]
Age	0.004	0.02	0.84	0.01	[−0.03, 0.04]	−0.02	0.02	0.36	−0.05	[−0.05, 0.02]
Gender	−0.05	0.15	0.72	−0.02	[−0.34, 0.24]	−0.11	0.14	0.44	−0.04	[−0.38, 0.16]
Education level	0.01	0.06	0.82	0.01	[−0.11, 0.14]	−0.10	0.06	0.10	−0.10	[−0.21, 0.02]
Household income	0.01	0.03	0.78	0.02	[−0.05, 0.07]	0.02	0.03	0.51	0.04	[−0.04, 0.08]
household size	−0.05	0.06	0.42	−0.04	[−0.16, 0.07]	−0.07	0.06	0.19	−0.07	[−0.19, 0.04]
Social desirability	0.12^**^	0.04	0.004	0.16	[0.04, 0.21]	−0.004	0.04	0.91	−0.01	[−0.08, 0.07]
Resilience	−0.15	0.08	0.052	−0.13	[−0.30, 0.001]	0.14^*^	0.07	0.050	0.13	[<0.001, 0.28]
Optimism	0.07	0.06	0.22	0.07	[−0.05, 0.19]	0.19^**^	0.06	0.001	0.21	[0.08, 0.30]
Gratitude	0.19^**^	0.06	0.001	0.25	[0.08, 0.31]	0.17^**^	0.06	0.002	0.25	[0.06, 0.28]
Joy	−0.13^*^	0.06	0.037	−0.14	[−0.26, −0.01]	0.002	0.06	0.97	0.003	[−0.12, 0.12]
Pride	−0.06	0.07	0.38	−0.07	[−0.19, 0.07]	−0.01	0.06	0.90	−0.01	[−0.13, 0.12]
Caring	0.04	0.07	0.62	0.04	[−0.10, 0.18]	0.08	0.07	0.20	0.10	[−0.05, 0.22]
Sadness	0.03	0.07	0.68	0.03	[−0.11, 0.17]	0.01	0.07	0.94	0.01	[−0.12, 0.14]
Anxiety	0.22^**^	0.07	0.001	0.26	[0.10, 0.35]	0.15^*^	0.06	0.010	0.19	[0.04, 0.27]
Anger	−0.05	0.07	0.48	−0.05	[−0.19, 0.09]	−0.11	0.07	0.10	−0.11	[−0.24, 0.02]

*Adjusted R^2^ of full model = 0.20, p < 0.001*.

*Adjusted R^2^ of full model = 0.21, p < 0.001*.

*ΔR^2^ due to gratitude = 0.03, p = 0.001*.

*ΔR^2^ due to gratitude = 0.03, p = 0.002*.

* *p < 0.05*,

** *p < 0.01. Gender was coded with “1” representing males and “0” representing females*.

Repeating the analyses on benefit-finding, the control variables explained significant variance in the first step (*R*^2^ = 0.23, *p* < 0.001), but gratitude significantly explained additional variance when entered in the second step (Δ*R*^2^ = 0.03, *p* = 0.002). The full model significantly explained variance in benefit-finding, *F*(16, 253) = 5.36, *p* < 0.001, Adjusted *R*^2^ = 0.21. Gratitude was again found to be an independent significant predictor of greater benefit-finding. Resilience and optimism both predicted greater benefit-finding. Anxiety significantly predicted greater benefit-finding whereas anger and sadness did not. Joy, pride, and care correlated positively with benefit-finding (Table 2), but these relationships were reduced to non-significant levels controlling for gratitude and other predictors. None of the demographic variables predicted benefit-finding.

## Discussion

Gratitude directs attention to the good things in one's life and widens our priorities to focus on others. As a result, it reduces the tendency to narrowly focus on a threat and the undesirable aspects in one's life. We hypothesized that gratitude should be associated with physically and psychologically beneficial responses during early stages of the COVID-19 pandemic, and report in this article likely the first empirical evidence consistent with our predictions. Chinese Singaporeans completed a survey during an uncertain period (March and April 2020) in which COVID-19 first emerged and escalated sharply in Singapore. The results showed that to the extent that the Chinese participants experienced gratitude, they were more likely to support virus-prevention measures and perceive meaningful benefits out of an adverse development. Another important finding is that these relationships held up even when controlling for known predictors of well-being and adjustment (resilience and optimism) and several other emotions, indicating the distinctiveness of gratitude in supporting healthy responses to the COVID-19 crisis. Social desirability was controlled for and a large sample of 417 participants were recruited, boosting the reliability of the findings. Several demographic variables that could predict the outcomes were also controlled for, indicating that the relationships are not attributable to them.

The findings suggest that gratitude could be a protective resource among Chinese people. Chinese people largely endorse collectivistic values that emphasize the inter-connectedness and the importance of serving not just the self but also others. Prior studies have found that attributes that are valued in a particular culture can be expected to produce stronger effects in that culture (34). Hence, we expected gratitude—a socially oriented positive emotion—to be uniquely associated with adaptive responses to the COVID-19 pandemic among the Chinese and the results support this contention in different ways. The relationships between gratitude and endorsement of virus-prevention measures and benefit-finding were not trivial but moderate in magnitude and remained robust controlling for a wide range of other potentially protective factors, emotion predictors, and demographic variables. In addition, among all emotions measured, gratitude was reported the most strongly. This finding was unexpected. Why were our participants more mindful of the good in their lives during the pandemic is unclear and deserves investigation in future research.

The finding concerning endorsing virus-prevention measures is consistent with the perspective that gratitude broadens and builds prosocial proclivities and openness to different prosocial methods (16). It suggests that gratitude can predict prosociality in a major crisis where many around the world are apprehensive about their own lives and livelihood. While the measures protect the self, they are fundamentally also meant to prevent the spread of an infectious virus and hence supporting them reflects a communal motivation to safeguard the physical well-being of others at some cost to the self. Further, the measures require a degree of openness to making significant changes in daily habits and personal preferences. The measures were difficult to accept when the pandemic started, when many people were not entirely convinced about their necessity or effectiveness. Hence, the finding is consistent with the idea that gratitude may prompt an openness to different and even untested ways to support the well-being of others (1, 16, 18). Note that prior research has rarely (if at all) tested whether gratitude may encourage the motivation to use unproven prosocial behavioral strategies in uncertain conditions—past studies that found links between gratitude and prosociality were largely conducted in crisis-free contexts and there is no research that directly demonstrated a link between gratitude and prosocial openness. In addition, the finding concerning benefit-finding further strengthens the idea that gratitude is associated with a flexible mindset that is open to different construals of events (18). It conceptually replicates prior findings that gratitude is linked to perceived coherence and positive appraisals of events (20), but also add to the literature in suggesting that gratitude is related to the ability to generate positive appraisals in highly adverse events.

While the other psychological predictors were included for testing the independent predictive power of gratitude, a short discussion on them is warranted. Resilience and optimism independently predicted greater benefit-finding but not stronger endorsement of the virus-prevention measures. There is replicable evidence that resilience is associated with stronger mental health (24), whereas evidence of a link between trait resilience and health-protecting behaviors appears sparse. In contrast, there is strong evidence that optimism is linked to both psychological well-being and engaging in positive health behavior (23). Hence, our finding on benefit-finding conceptually replicates past work but more research is needed on whether resilience and optimism are linked to health-protecting responses to the COVID-19 pandemic. With the exception of anxiety, the other emotions did not independently predict both outcomes positively, casting doubts on whether they could be psychological resources that enable crisis coping. It could be that the anxious participants were more willing to use preventive measures because they helped to reduce the uncertainty that COVID-19 elicits and protect themselves from getting infected, which is consistent with the function of anxiety to avoid threats. Strangely, anxiety also predicted greater benefit finding. We speculate that this is because to the more anxious participants, perceiving the crisis in more positive angles was a useful coping strategy that enabled them to manage their distress. However, recent studies found that anxiety due to COVID-19 predicted the use of both negative and positive coping mechanisms (35) and impaired daily work functions and relationships (36). Hence, it is still unclear whether anxiety is linked to positive or negative coping responses to the COVID-19 crisis.

The findings suggest that gratitude does not merely predict prosociality. Rather, gratitude may predict a greater form of prosociality that makes the grateful person open to a range of means to help others and serve the community, including means that may compromise personal needs and wants (1, 16). The findings may also suggest that gratitude can increase receptiveness to advices to health experts on the scientifically supported ways to reduce spread of the virus. A key next step is to test the robustness of our findings. Another step is to test the extent of self-sacrificial prosocial behavior that gratitude might encourage in a health crisis—e.g., would it prompt individuals to make money, time, and blood donations? Furthermore, considering the greater openness of grateful individuals, it is also pertinent key to test whether they would be receptive to wrong advices, given the volume of misinformation circling in the media today.

### Limitations

First, the findings are cross-sectional and we make no causal claims. The gratitude items referenced past feelings and the virus-prevention measures directed participants' attention to the future, and hence an argument could be made that the direction of causality should be from gratitude to virus-prevention measures. Hence, there is still a need for studies that manipulate and test the causal effects of gratitude on the current outcomes. Second, meta-analytic research found only small effects of gratitude interventions and the effects varied with specific outcomes and control conditions (37, 38). Hence, even if experimental evidence of the effects of gratitude becomes available, much additional work would be required to validate its effectiveness as an intervention strategy in enabling individuals to cope with the COVID-19 pandemic. Third, another limitation is that it is unclear what might mediate the relationship between gratitude and the outcome variables. Based on the theoretical considerations outlined in this article, we expect that prosocial intention and cognitive openness are likely mediators—future studies may test these mediators. Fourth, the current study was conducted when the pandemic started. More research is needed on whether gratitude continues to play protective functions now when people around the world have lived with the pandemic and all its negative effects for months. Finally, more research would be needed to test whether the findings replicate in non-Chinese samples and also other Chinese groups.

## Conclusion

Given the limitations, we take a circumspect approach to interpreting the generalizability of the finding. However, the findings suggest that gratitude could be a valuable coping resource among Singaporean Chinese. Specifically, gratitude is linked to a greater intention to use protective measures that can slow the spread of COVID-19 to support community health and finding constructive meaning during the crisis. It is also unique among other emotions and protective factors in supporting these responses. Implications for policy-makers and practitioners would be to encourage individuals to avoid focusing excessively on the threats and losses that the pandemic brings and direct their attention toward positive things in their lives that they can be grateful for.

## Data Availability Statement

The datasets presented in this study can be found in online repositories. The names of the repository/repositories and accession number(s) can be found in: https://osf.io/k793q/?view_only=8757f08d2e354c76a857de1b052694ef.

## Ethics Statement

This study has been approved by the IRB of the National University of Singapore. All participants provided consent before participating in this study.

## Author Contributions

ET conceptualized the research. Both authors wrote the paper. VO collected and analyzed the data. Both authors contributed to the article and approved the submitted version.

## Conflict of Interest

The authors declare that the research was conducted in the absence of any commercial or financial relationships that could be construed as a potential conflict of interest.

## Footnotes

^1^Participants rated the emotion items without a specific target. Hence, general forms of the emotions including gratitude were measured. Accordingly, the ensuing findings are of greater generalizability because they describe how gratitude in general (rather than specific forms of gratitude) is related to the outcomes.
